# Long-Term Survival Following Palliative Chemoradiotherapy in an Elderly Patient With Advanced Squamous Cell Carcinoma in the Right Mandibular Gingiva

**DOI:** 10.7759/cureus.22142

**Published:** 2022-02-11

**Authors:** Yojiro Ishikawa, Rei Umezawa, Takaya Yamamoto, Kengo Ito, Keiichi Jingu

**Affiliations:** 1 Department of Radiation Oncology, Tohoku University Graduate School of Medicine, Sendai, JPN; 2 Division of Radiology, Tohoku Medical and Pharmaceutical University, Sendai, JPN

**Keywords:** abscopal effect, squamous cell carcinoma, s-1, radiation therapy, mandibular gingival cancer

## Abstract

Advanced squamous cell carcinoma (SCC) of the mandibular gingiva in elderly patients is difficult to cure. The treatment policy for elderly patients with advanced SCC of the mandibular gingiva has not been clear. We report a case of right mandibular gingival carcinoma that was successfully treated by palliative chemoradiotherapy. An 83-year-old female complained of pain and an ulcer in her right mandibular gingiva. Oral examination revealed a lesion of about 20 mm in size in the right mandibular gingiva. A diagnosis of SCC in the right mandibular gingiva was made by histology. Imaging findings revealed some right neck lymph node metastases. Based on these findings, a clinical diagnosis before treatment was SCC in the right mandibular gingiva (cT4aN2bM0, stage IV) by the 7th edition of the Union of International Cancer Control. She refused to receive definitive surgery or chemoradiotherapy due to concerns about the invasiveness of these definitive therapies and requested palliative chemoradiotherapy. We delivered S-1 (a combination of tegafur, gimeracil, and oteracil) and radiation therapy (RT) to the primary tumor alone with 30 Gy in 10 fractions using 4-megavoltage equipment via a multiple leaf collimator by three-dimensional RT. Although we could not complete the delivery of S-1 because of an acute side effect, the palliative chemoradiotherapy resulted in a complete response, and the lymph node metastases also disappeared. The patient remains in complete remission for 5 years without surgery or chemotherapy. Palliative chemoradiotherapy for elderly patients with mandibular gingival carcinoma is considered to be one of the therapeutic options.

## Introduction

Oral cancer is a difficult malignant neoplasm to cure. Squamous cell carcinoma (SCC) is a common type of oral cancer [1]. Approximately 10% of all malignant tumors of the oral cavity occur on the gingiva [2]. The National Comprehensive Cancer Network guideline and previous reports recommend that treatments for the mandibular gingiva of SCC are primarily surgical. Radical neck dissection is the standard treatment for metastatic lymph nodes [3-4]. However, the prognosis of advanced SCC of the mandibular gingiva is poor [5], palliative radiation therapy (RT) is usually the preferred modality of treatment for advanced cancer of poor performance status (PS).

In the current aging society, not all patients can be treated with surgery because of their clinical condition or complications. However, the treatment strategy for patients of advanced age who cannot receive definitive therapy is unclear. We herein report the achievement of long-term survival after palliative RT with S-1 (a combination of tegafur, gimeracil, and oteracil) for SCC of the mandibular gingiva. This is the first report of a patient with long-term survival palliative chemoradiotherapy for SCC of the mandibular gingiva. This article was previously uploaded as a preprint on Research Square [6].

## Case presentation

**Figure 1:**
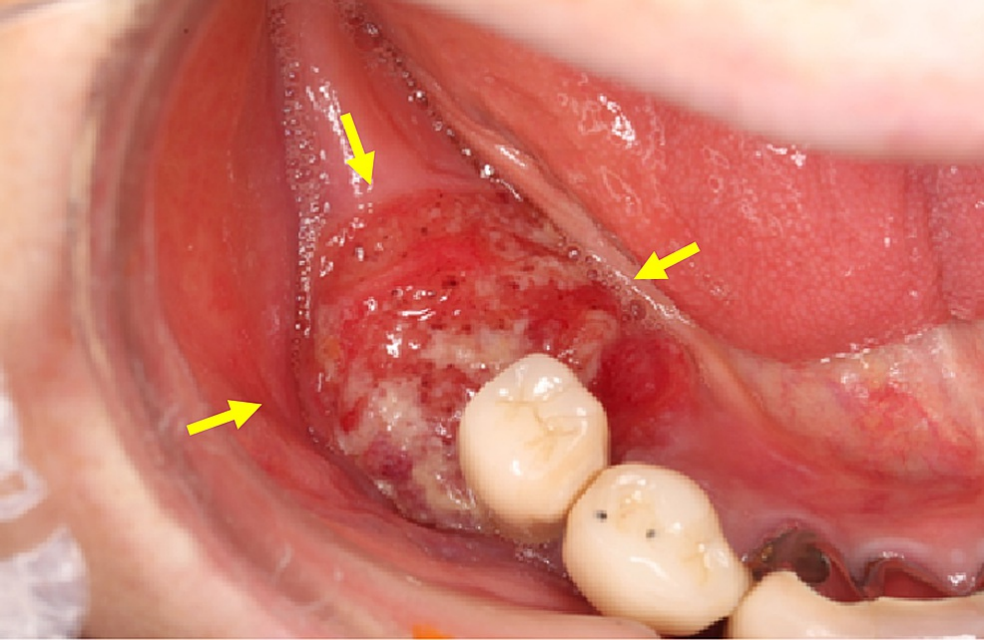
Oral findings Oral examination revealed a lesion of 20 mm in size in the lower right gingiva (yellow arrow).

An 83-year-old Japanese woman complained of pain and an ulcer in her right mandibular gingiva. Her dentist adjusted her denture, but the lesion in the right mandibular gingiva did not improve and she was recommended to receive an examination in a hospital. The patient had a history of alcohol consumption (approximately 350 mL of beer per day for 20 years) but no smoking. The medical history of the patient was hepatitis C, diabetes mellitus, and hyperlipidemia. Her medications were metformin, sitagliptin phosphate hydrate, and rosuvastatin calcium. Her family history included liver cancer in her father. Oral examination revealed a lesion of 20 mm in size in the lower right gingiva (Figure 1).

**Figure 2:**
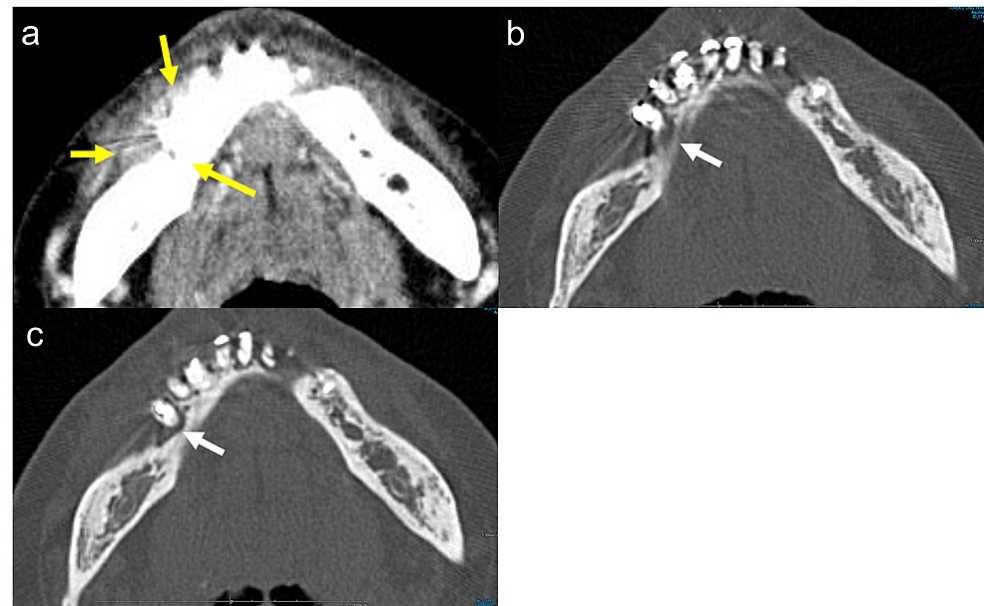
Axial-enhanced CT Axial-enhanced CT scan images of the mandible showed an enhanced tumor that had spread from the right mandibular gingiva to the right lower mandible (yellow arrow) (a). The lesion destroyed the alveolar bone (white arrow) (b). There was also bone resorption around the adjacent teeth (white arrow) (c).

Computed tomography (CT) revealed a tumor that had spread from the right mandibular gingiva to the right lower mandible. The lesion had destroyed the alveolar bone and there was a possibility of periodontal disease or gingival cancer (Figure 2).

**Figure 3:**
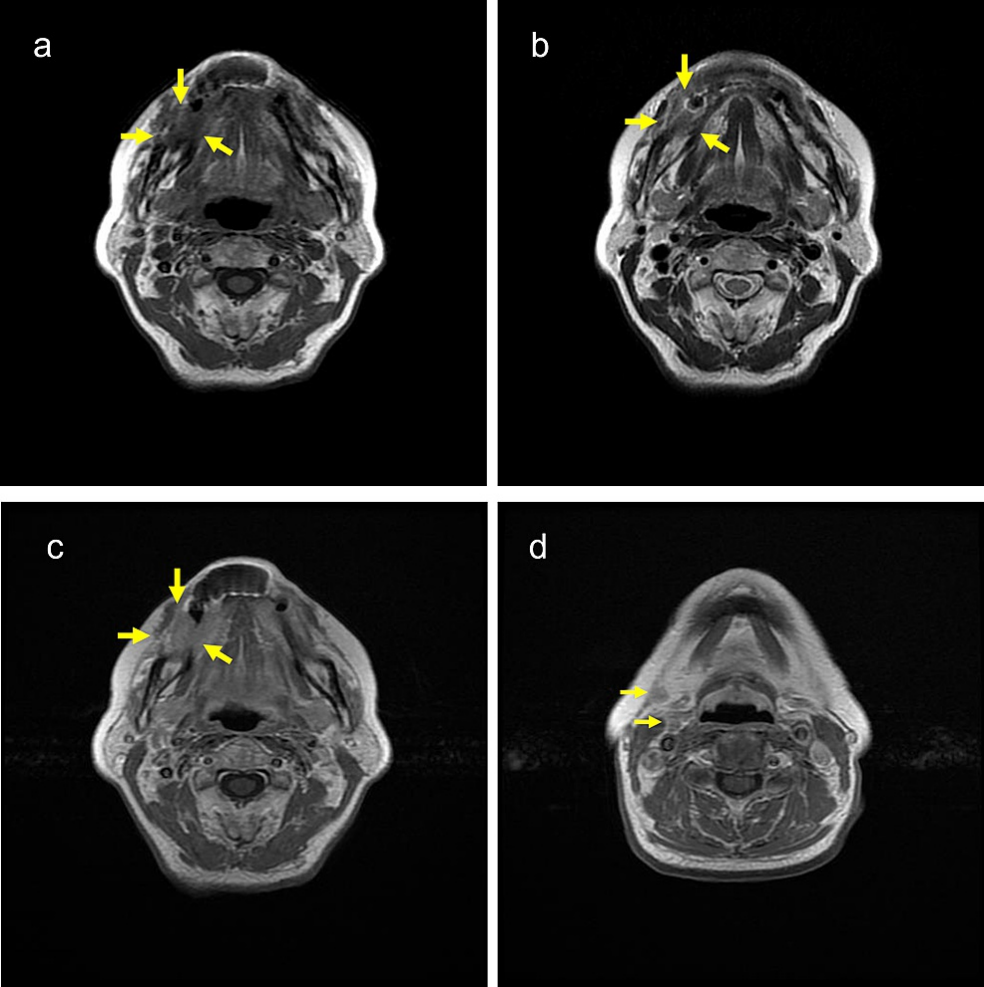
Axial MRI of the head and neck MRI showed an irregular tumor in the lower right gingiva. The tumor showed lower intensity on a T1WI (yellow arrow) (a) and intermediate intensity on a T2WI (yellow arrow) (b). A gadolinium-enhanced T1WI, a uniform enhancing tumor extended to the mandible bone (yellow arrow)(c). A gadolinium-enhanced T1WI shows two small lymph nodes of the right neck (yellow arrow) (d).

Magnetic resonance imaging (MRI) revealed an irregular tumor in the lower right gingiva and two enraged lymph nodes in the right neck region. The tumor showed lower intensity on a T1-weighted image (WI). The tumor showed intermediate intensity on a T2WI and the mandible bone close to the tumor showed diffuse lower intensity. On a gadolinium-enhanced T1WI, a uniform enhancing tumor extended to the mandible bone (Figure 3).

**Figure 4:**
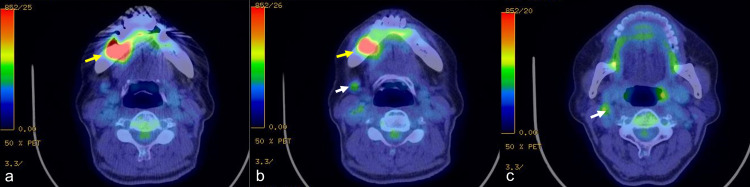
Positron emission tomography Positron emission tomography-CT showed uptake of 18F-2-fluoro-2-deoxy-D-glucose (FDG) in the mandibular gingiva with a maximum standardized uptake value (SUVmax) of 14.5 (yellow arrow) (a-b). Uptake of FDG of two lymph nodes of the neck had an SUVmax of 2.0-2.3 (white arrow) (b-c).

Positron emission tomography-CT (PET-CT) revealed uptake of 18F-2-fluoro-2-deoxy-D-glucose (FDG) on the right mandibular gingiva (maximum standardized uptake value (SUVmax) of 14.5) and on two lymph nodes in the right neck region (SUVmax of 2.0-2.3) (Figure 4).

We could not confirm the pathological diagnosis for lymph node metastases. The diagnosis based on histological examination of biopsy specimens from the lesion was SCC. The clinical diagnosis before treatment was SCC in the right mandibular gingiva (cT4aN2bM0, stage IV). We used the tumor, nodes, and metastases (TNM) classification 7th edition by the Union of International Cancer Control (UICC) for cancer staging.

Although surgery is the standard therapy for SCC of the mandibular gingiva, the patient refused to receive surgery due to concerns about damage after surgery. She and her family requested palliative dose radiotherapy and oral medication of chemotherapy for her pain of right mandibular gingiva.

**Figure 5:**
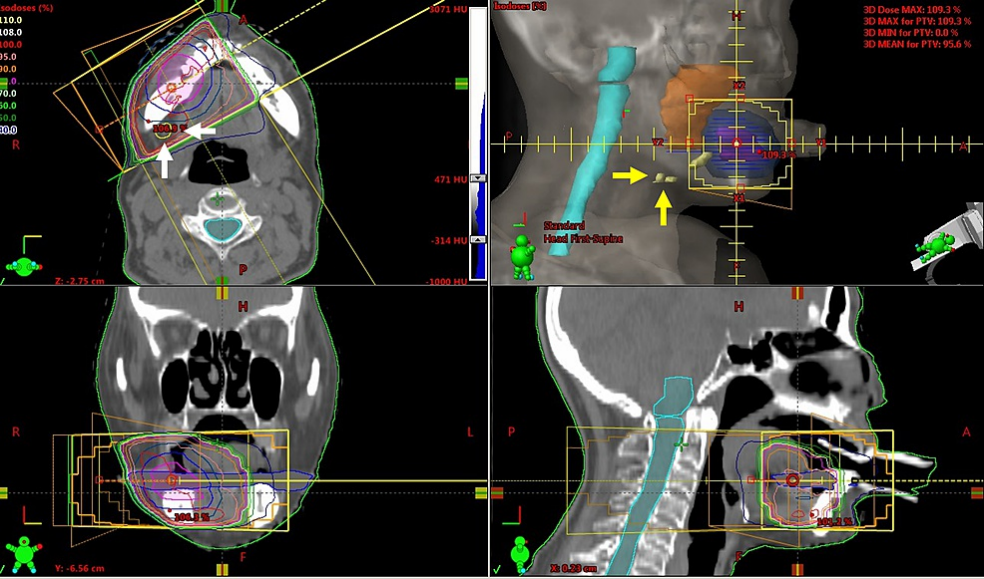
Radiation therapy The primary carcinoma of the right mandibular gingiva received 30 Gy in 10 fractions using 4 megavoltage equipment via a multiple leaf collimator by three-dimensional radiation therapy. A lymph node metastasis received 15-30 Gy (white arrow), another lymph node did not receive radiation (yellow arrow).

We delivered radiation therapy RT of 30 Gy in 10 fractions over a period of 2 weeks with S-1 (a combination of tegafur, gimeracil, and oteracil). RT was delivered with 4-megavoltage equipment via a multiple leaf collimator by three-dimensional RT (Figure 5).

**Figure 6:**
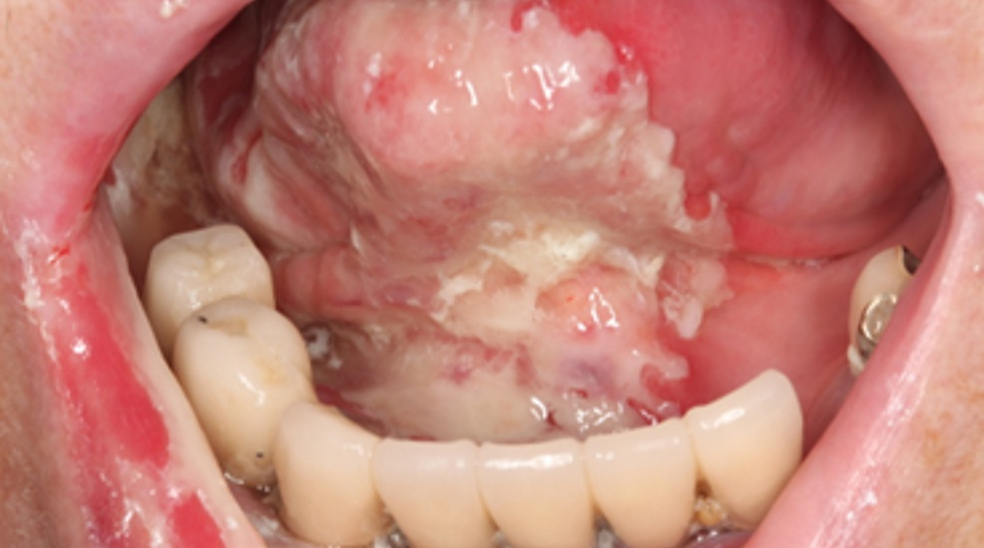
Oral findings (acute side effect) Acute side effect of grade 3 stomatitis according to the National Cancer Institute Common Terminology Criteria for Adverse Events version 4.0. Mucositis in the oral cavity was more severe on the right side, which coincided with the irradiation field. It was more severe than the usual mucositis that occurs at 30 Gy.

Gross tumor volume (GTV) was defined as the primary tumor alone based on pretreatment examination of CT, MRI, and PET-CT. We did not contain right cervical lymph node metastases because these metastases did not associate with her pain of right mandibular gingiva. The clinical target volume (CTV) was defined as GTV plus 0.5-cm margins. The planning target volume (PTV) was CTV plus 0.5-cm margins (Figure 6).

On the 12th day of treatment, we canceled the administration of S-1 because the patient had an acute side effect of grade 3 stomatitis and fatigue by chemoradiotherapy according to the National Cancer Institute Common Terminology Criteria for Adverse Events version 4.0. Mucositis in the oral cavity was more severe on the right side, which coincided with the irradiation field. It was more severe than the usual mucositis that occurs at 30 Gy (Figure 6). As a result of loss of appetite due to severe stomatitis, the patient was hospitalized for 1 week.

**Figure 7:**
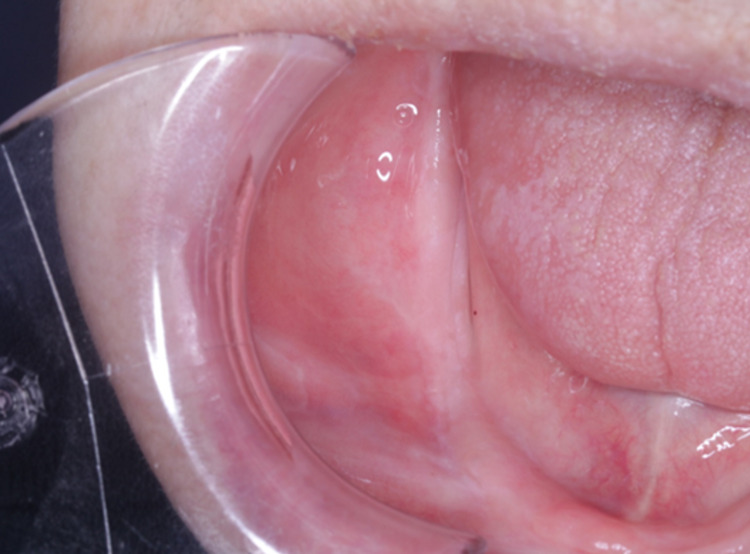
Oral findings 5 years after the chemoradiotherapy The patient remains in complete remission 5 years after the initial treatment without surgery or chemotherapy.

After treatment, imaging showed no evidence of recurrence and the lymph node metastases that had not been irradiated had also disappeared. The patient remains in complete remission (CR) for 5 years after the initial treatment without surgery or chemotherapy (Figure 7).

## Discussion

Carcinomas of the mandibular gingiva are more common than those of the maxillary gingiva [7]. Mandibular gingival carcinoma invades the mandible in an early stage [8]. If it invades the mandible deeply, it cannot be expected to be cured by RT alone because radiation osteoradionecrosis occurs after high-dose RT [9]. In addition, the transfer of antitumor drugs to bone is poor and surgical resection is performed for carcinomas of the mandibular gingiva [4].

In stage III or IV, locally advanced SCC of the head and neck is usually treated by surgery or concurrent chemoradiotherapy [10-12]. The 5-year overall survival (OS) rate after resection for mandibular gingival carcinoma has been reported to be 38-80.6% [4,5]. On the other hand, the 5-year disease-free survival rate of patients with cervical metastasis is about 25% [13]. In the present case, good control was achieved without resection. The combination of RT and S-1 might have been good. S-1 is an oral anticancer drug-containing gimeracil and oteracil potassium in tegafur that is a prodrug of 5-fluorouracil (5-FU). Gimeracil inhibits dihydropyrimidine dehydrogenase, which is a 5-FU-degrading enzyme, and oteracil potassium has the effect of suppressing gastrointestinal toxicity caused by 5-FU [14]. Gimeracil was reported to exert radiosensitizing effects on oral SCC in vitro and in vivo [15]. Response rates of 46.2% and 28.3% were reported for early and late phase II clinical trials for advanced and recurrent head and neck cancer, respectively [16]. There are also some reports of CR with the administration of a single agent for head and neck cancer and oral cancer [17]. In addition, elderly patients are considered to be tolerant of S-1 [18].

A severe acute complication occurred in our patient. It is desirable to reduce the side effects of chemotherapy as much as possible. Alternate day administration of S-1 may help to reduce side effects. This takes advantage of the fact that the cell cycle in humans is about one day, whereas that of cancer cells is 5-7 days [19,20]. In animal experiments, it was shown that the effects were the same and that side effects were reduced compared to alternate-day administration and daily administration [19].

The S-1 might have burned out the lymph node metastases in our case. There are some reports that CR was obtained only with S-1 [17]. In the present case, the combined use of S-1 is only for 1 week, and the effect of S-1 alone is considered to be limited. It was possible that the reactive swelling has improved because the ulcer in the primary lesion has disappeared. In addition, we could not confirm the pathological diagnosis for lymph node metastases. It is possible that it was a misdiagnosis for nodal clinical staging. The staging of oral cancer has been changed in recent years, and it is possible that the stage of this case was overestimated. For tumors of oral cancer, the most recent 8th edition of the UICC staging system has introduced a depth of infiltration (DOI) as a novel parameter. The DOI in our case was less than 10 mm and the tumor was more than 20 mm, which is considered to be equivalent to cT2N2bM0 by the 8th edition of the UICC. The abscopal effect is probably associated with our local control for lymph node metastases in the right neck region out of the radiation field. This effect is known that radiation shrinks tumors outside the irradiation field [21]. The biological mechanism underlying this effect remains unclear. To the best of our knowledge, there has been no study on the abscopal effect of mandibular gingival carcinoma. In our case, it is difficult to distinguish between the effects of chemotherapy and the potential for abscopal effects.

Patients with head and neck cancer who receive cumulative radiation doses of more than 50 Gy are more likely to have oral side effects and they have a higher risk of unplanned breaks in RT [22]. In palliative RT, regimens for elderly patients or poor PS, Grewal et al. recommended that 44 Gy in three cycles (3.7 Gy delivered twice daily for 2 consecutive days), 21 Gy in three fractions, or 20 Gy in five fractions. In addition, 30-50 Gy in 10-25 fractions of palliative RT for good PS were recommended [23]. Our patient was hospitalized for 1 week due to loss of appetite and an oral side effect, but the low radiation dose of 30 Gy with S-1 might have been a treatment option for elderly patients with advanced carcinoma in the mandibular gingiva.

## Conclusions

We reported the achievement of long-term survival after palliative RT with S-1 (a combination of tegafur, gimeracil, and oteracil) for SCC of the mandibular gingiva. This report is an interesting case of a patient with long-term survival palliative chemoradiotherapy for advanced SCC in mandibular gingiva. Because this report is a case study, it is difficult to define the indication for palliative chemoradiotherapy in an elderly patient with advanced SCC in mandibular gingiva. However, it is possible that some patients with advanced SCC in mandibular gingiva were treated only by surgery, chemotherapy, or RT although they were potential candidates of palliative chemoradiotherapy. Palliative chemoradiotherapy for advanced SCC in mandibular gingiva is considered to be one of the therapeutic options.
